# Screening criteria: the need to deal with new developments and ethical issues in newborn metabolic screening

**DOI:** 10.1007/s12687-012-0118-9

**Published:** 2012-10-07

**Authors:** John Forman, Fiona Coyle, Jill Levy-Fisch, Pat Roberts, Sharon Terry, Michael Legge

**Affiliations:** 1 New Zealand Organisation for Rare Disorders (NZORD), PO Box 38-538, Wellington Mail Centre, 5045 Wellington, New Zealand; 2 Save Babies Through Screening Foundation, PO Box 42197, Cincinnati, OH 45242 USA; 3 Save Babies Through Screening Foundation, UK, 22 Bransdale Avenue, Guiseley, Leeds, LS20 8QA UK; 4 Genetic Alliance INC, 4301 Connecticut Ave. NW, Suite 404, Washington, DC 20008-2369 USA; 5 Department of Biochemistry, University of Otago, PO Box 56, Dunedin, New Zealand

**Keywords:** Newborn metabolic screening, Decision criteria, Family interests

## Abstract

Newborn metabolic screening is the most widespread application of screening technology and provides the most comprehensive application of genetics in health services, where the Guthrie blood spot cards allow screening for metabolic diseases in close to 100 % of all newborn babies. Despite over 40 years of use and significant benefits to well in excess of 100,000 children worldwide, there is remarkably little consensus in what conditions should be screened for and response to new advances in medicine relating to programme expansion. In this article, the international criteria for newborn metabolic screening are considered, and we propose that these criteria are poorly developed in relation to the baby, its family and society as a whole. Additionally, the ethical issues that should inform the application of screening criteria are often not developed to a level where a consensus might easily be achieved. We also consider that when family interests are factored in to the decision-making process, they have a significant influence in determining the list of diseases in the panel, with countries or states incorporating family and societal values being the most responsive. Based on our analysis, we propose that decision criteria for metabolic screening in the newborn period should be adapted to specifically include parent and family interests, community values, patients’ rights, duties of government and healthcare providers, and ethical arguments for action in the face of uncertainty.

## Introduction—the context of pregnancy, childbirth and neonatal screening

Newborn metabolic screening is a distinct subset of the varied screenings that are available in the prenatal and neonatal period. Maternity care revolves around many screens for maternal infections, blood pressure, gestational diabetes, fetal abnormalities and other risks to the mother and fetus. The identification of such risks permits a range of interventions to prevent serious health problems for mother and baby throughout the pregnancy and birth process. Furthermore, after birth there are screening options for hearing loss (White et al. 1994; Yoshinaga-Itano 2004), metabolic diseases (Garg and Dasouki 2006; Yoon et al. 2005) and other physical disorders (Fisher 1991; Pass et al. 2000; Quinn et al. 1977).

Public health screening programmes are rare occurrences in maternity care, with non-programme screening being a more common practice. Referred to as ‘opportunistic screening’ or ‘standard medical practice’, the health professional evaluates and tailors the tests to the patient’s individual circumstances. In contrast, other screening tests are offered to all people falling into a particular category, as part of a planned public health programme, which are largely based on the ten principles proposed by Wilson and Jungner (1968) primarily formulated in relation to cancer detection; however, since that report additional criteria have been considered in the context of medical and screening practice in the twenty-first century (Andermann et al. 2008). These programmes have significant implications, both for individuals offered tests and for health systems in general. As discussed below, there are detailed analyses against criteria for screening programmes, including cost benefits and assessment of potential benefits and harms, and programme standards and quality measures, before such programmes are established. More recently, there have been moves to introduce new forms of screening which are specifically pregnancy and child birth-related into formal public health programmes. This includes antenatal HIV, antenatal fetal aneuploidy and newborn hearing tests. However, the most universally accepted and long-standing programme in most developed countries is newborn metabolic screening. Overall, these are well-run programmes with little harm to the newborn; however, it is our belief that the use of the screening programmes could be more effective if broader considerations are given to the overall welfare of the family and the overall principles proposed by Andermann et al. (2008) as well as the identification of a specific disease in the newborn. Here, we will consider the background of newborn metabolic screening in the context of benefit in relation to respect for autonomy, ethical conduct and choice within the family.

## Newborn metabolic screening programme: a short history

Newborn metabolic screening evolved from Guthrie and Susi (1963) test for metabolites from dried blood spots. Using a bacterial inhibition assay whereby the growth of *Bacillus subtilis* is enhanced in the presence of phenylalanine, he was able to identify babies with phenylketonuria (PKU) prior to clinical presentation. As is common in most metabolic disorders, once PKU symptoms are apparent, cellular damage has already occurred. Newborn blood test screening permits early recognition and enables dietary intervention to prevent the severe mental retardation that would inevitably occur as a consequence of the enzyme phenylalanine hydrolase deficiency or mutations in the enzyme (Hansen 1975; Walter 1998). The ‘PKU test’, as it is known, has been embraced by all modern health systems and is widely regarded as an exemplar of a successful public health screening programme.

Later, an increase in knowledge and technology allowed for the testing of an increasing number of diseases from the same blood spots (Clague and Thomas 2002). For instance, starting in the 1970s (1981 in New Zealand), congenital hypothyroidism (CH) has been widely adopted by screening programmes (Ehrlich and McKendry 1973; Fisher 1991; National Testing Centre 2010; Taranger et al. 1973). The test detects thyroid-stimulating hormone deficiency, allowing early treatment to prevent the onset of severe physical and mental deterioration.

Generally, beyond this consensus on the use of PKU and CH screening programmes, the adoption of other tests is geographically variable (Clague and Thomas 2002). For example, in New Zealand, galactosemia, congenital adrenal hyperplasia, biotinidase deficiency, cystic fibrosis (CF) and maple syrup urine disease were successively added to the list of screening conditions, over the 1970s and 1980s (National Testing Centre 2010). In other countries, opportunities were taken to add additional tests to the screening programme such as haemoglobinopathies as a result of high carrier rates in specific populations (Benson and Therrell 2010; Streetly and Dick 2005). However, prior to expanded screening on an international basis, the number of tests in each health system or US state ranged from as few as two or three up to seven (Watson et al. 2006). During the1990s, advances in technology led to the development of tandem mass spectrometry, with the capacity to accurately screen for a much larger number of rare metabolic diseases (Hill 1993; Jones and Bennett 2002; Röschinger et al. 2003). By 2007, screening was underway for an average of about 27 metabolic disorders throughout most US states, parts of Canada and all of Australia and New Zealand (Sharrard and Pollitt 2007). In contrast, despite a modest increase in screening targets in Britain and other parts of Canada, there are still considerably fewer tests offered by so-called expanded screening programmes. There is substantial literature that is either supportive (Tarini 2007; Avard et al. 2007; Lin and Fleischman 2008; Alexander and van Dyck 2006; Howell 2006) or critical/cautious about expanded newborn screening (Bailey and Murray 2008; Moyer et al. 2008; Grosse et al. 2006; Botkin et al. 2006). Internationally, some jurisdictions are noted for their prompt uptake of the associated technologies, with others slow and seemingly reluctant to follow the trend (Green et al. 2006; Padilla et al. 2010).

Adding to the contention about further expansion of screening is debate about how to respond to technological advancement that makes it technically possible to screen for Fragile X (Bailey and Murray 2008; Coffee et al. 2009), lysosomal storage diseases (Li et al. 2004; Meikle et al. 2006), immune deficiencies (Cassol et al. 1994; Puck 2007), Duchenne muscular dystrophy (Parsons and Bradley 2008; van Ommen and Scheuerbrandt 1993) and other rare disorders (Röschinger et al. 2003).

Differentials in the uptake of disorders into screening programmes are suggestive of discrepancies between screening criteria and a lack of international standardization (Tuuminen et al. 1994). The development of screening programmes and the differences that have evolved are the consequence of context-specific interpretations of and amendments to screening criteria (Clague and Thomas 2002; Padilla et al. 2010). Moreover, they are also dictated by financial resources, incidence rate, the strength of patient advocacy and cultural differences (Pollitt 2007).

## Screening criteria

Screening criteria originated from a well-cited paper by Wilson and Jungner (1968) that was commissioned by the World Health Organisation (WHO). Wilson and Jungner’s criteria were primarily formulated in the context of screening for adult diseases specifically carcinomas and hepatitis B (Table 1). The authors’ intention was for the criteria to be adapted and developed within differing situations, as opposed to strict adherence to a formula. However, in practice, many health systems appear to regard them as static, rather than an evolving regime. They are frequently referred to as a ‘gold standard’ for screening (Andermann et al. 2008). Although Wilson and Jungner’s criteria have undergone some refinement to incorporate issues such as the validity of tests (Cochrane and Holland 1971), they nevertheless remain as a set of criteria that have attained a state of almost biblical reverence for many commentators.

**Table 1:** The principles proposed by Wilson and Jungner (1968) for the early detection of disease

1.	The condition sought should be an important health problem.
2.	There should be an accepted treatment for patients with recognized disease.
3.	Facilities for diagnosis and treatment should be available.
4.	There should be a recognizable latent or early symptomatic stage.
5.	There should be a suitable test or examination.
6.	The test should be acceptable to the population.
7.	The natural history of the condition, including development from latent to declared disease, should be adequately understood.
8.	There should be an agreed policy on whom to treat as patients.
9.	The cost of case finding (including diagnosis and treatment of patients diagnosed) should be economically balanced in relation to possible expenditure on medical care as a whole.
10.	Case finding should be a continuing process and not a “once and for all” project.

However, this poses difficulties when attempts are made to impose the criteria in the context of dissimilar disease categories, such as newborn metabolic screening. Indeed, Wilson and Jungner noted that it was at an early developmental phase at the time, and consequently did not factor newborn metabolic screening into the development of their criteria (Wilson and Jungner 1968). In contrast to cancer screening, situations such as newborn screening for a range of diseases are distinct in their nature. For instance, the newborn baby lacks the autonomy of an adult who decides to undergo screening for cancer. Instead, these decisions are made by and directly impact upon the baby’s parents, an additional complication that needs special consideration. Despite this, there has been no international consensus on an appropriate set of criteria for the newborn context (Clague and Thomas 2002; Padilla et al. 2010; Tuuminen et al. 1994). In order to explore how these difficulties are managed in practice, we now turn to a specific case study: New Zealand.

## Screening criteria in New Zealand: the case of cystic fibrosis

New Zealand was an early adopter of newborn metabolic screening and set up one of the first national screening programmes for all newborns in 1969 (National Testing Centre 2010). In 2003, the National Health Committee (NHC) updated their assessment criteria for health screening programmes in New Zealand. The NHC document outlines five components that constitute what they term a ‘quality’ programme: safety, consumer focus, access, effectiveness and efficiency. Screening assessments criteria are also identified that are consistent with the WHO formula, albeit with the addition of social, ethical and cost–benefit considerations (National Health Committee 2003).

Although these criteria appear to be robust, there is little reference to the context of newborn screening; in particular, how the formula should be applied in practice. With a primary analysis of the screening scenarios of four types of cancer and hepatitis B, the report makes only two references to newborn screening. The first reference is in a list of examples of screening in New Zealand; the second is a brief comment on the ethical issues surrounding the consent process in relation to screening children. With an absence of guidance on how to implement the screening criteria in the practice of newborn screening, some interpretation and flexibility in applying them is both needed and used. To demonstrate this, we explore how this has occurred at ground level in the context of screening for CF.

CF is a disease that leads to increasing disability and in many cases, early mortality (Ramsey 1996). Whilst it affects the entire body, the most common symptom is breathing difficulties that result from frequent lung infections and increased secretions. Other symptoms include poor growth, sinus infections, diarrhoea, scarring of the pancreas and infertility. It is an autosomal recessive mutation in the cystic fibrosis transmembrane conductive regulator gene resulting in abnormal regulation of the components of mucus, sweat and digestive enzymes (Bush and Gotz 2006).

Following work by Crossley et al. (1979) at the University of Auckland, cystic fibrosis was introduced as a research project into the New Zealand newborn metabolic screening programme in 1983. However, the Ministry of Health was reluctant to provide for its continuation. Whether the Ministry’s reasons were based on compliance with screening criteria, on cost, on cost effectiveness based on outcomes for the child, or all of these combined is not clear, but following significant support group lobbying, a decision to retain the project on a permanent basis was made at a political level.

Whilst cystic fibrosis did not strictly adhere to the WHO screening criteria, the crux of the argument for continued inclusion in the newborn screening programme revolved around early identification and early intervention, including family knowledge of inheritance risk. Consequently, whilst there was, and still is, no fully effective treatment for cystic fibrosis, newborn screening has enabled early intervention in 354 New Zealand cases (National Testing Centre 2010) that occurred from 1983 to December 2010. There is uncertainty about the individual contributions of each factor, but it is clear that the combined effect of early detection and intervention, and treatment advances, permits patients who would invariably die as children to live well into adulthood. Cystic fibrosis, largely as a result of screening, but also helped by improved medicines, has been transformed from a disease that was usually fatal in childhood to a manageable chronic disease (Bush and Gotz 2006).

Moreover, incorporating cystic fibrosis into the New Zealand newborn screening programme was a landmark event, one where screening is implemented despite the fact that the condition does not strictly interface with the official criteria. More recently, research from Australia and elsewhere has shown good clinical benefit from screening, and it is now being implemented throughout North America and other countries and states (Green et al. 2006).

The cystic fibrosis case highlights aspects of decision making that are not anticipated in the WHO or New Zealand screening criteria. Evidence of improved outcomes to the existing natural progression of the diseases was not certainly known in advance of the screening that allowed those improvements to occur. The experience of treating physicians was an important consideration, along with the support of advocacy groups keen to improve health outcomes for families.

Thus, it seems that in ground-level situations, a pragmatic ethic adopted by healthcare systems can overrule the pre-established framework stemming from the original WHO or New Zealand screening criteria. But what does this tell us about those criteria? In the next section, we utilize the ground-level experience and decision making to critique aspects of the WHO and New Zealand screening criteria.

## Ethical frameworks for newborn screening decisions

The ‘Four Principles’ medical ethics framework (Beauchamp and Childress 2001) is widely accepted at an international level, and offers a broad consideration of issues within the medical ethics field. This is not unexpected, for the framework highlights principles that are highly relevant to the field of medicine: respect for autonomy, beneficence, avoiding harm and justice. Although, in theory, the WHO and New Zealand screening criteria comply well with it, in practice, their application matters a great deal. For example, if benefits and harms are applied as though to an adult, one outcome may result; another outcome may emerge from these principles as applied to a newborn baby if the interests of this young child are seen as intertwined and perhaps inseparable at that stage of life from the close interests of parents and family. Benefits to a family might be an indirect but still significant benefit to the newborn (Bailey et al. 2005; Burchbinder and Timmermans 2011; Wilcken 2012). In order to tease out the complexity of the ethical issues that arise in practice, and consider how they might be appropriately applied, we now briefly focus on some concrete examples from the New Zealand context. These principles, derived from this context, directly contrast with the criteria outlined in the Wilson and Jungner formula, and we examine the processes by which they may be weighed up and implemented, in contradiction to standard procedures.

## Screening for conditions where the evidence is uncertain or unavailable

Globally, it is estimated that there are 6,000 to 8,000 different rare disorders that have prevalence of less than 1 per 2,000 people in the European population or fewer than 200,000 people in the USA (European Commission Position Statement on Rare Diseases and Orphan Drugs 2010). The subsequent lack of an evidence base for rare disorders is thus a sticking point when it comes to the seventh criterion outlined by Wilson and Jungner, which pivots around an emphasis on screening for diseases that are ‘adequately understood’. It also raises the issue of finding a balance between benefits and harms. All of the conditions that are currently in the newborn metabolic screening programme are rare, as are the candidates for subsequent inclusion. A ‘comprehensive natural history’ of rare disorders is often not available, and it may be unethical or impossible to attempt controlled trials in such severe diseases when treatment or other intervention has become available.

Even the highly successful PKU programme had some benign forms picked up when that programme started, giving rise to false positive results. This resulted in some associated harms such as unnecessary parental anxieties and the restriction of protein in the diet of a growing child, and action was required to adapt the programme and management of those identified (Gurian et al. 2006; Hewlett and Waisbren 2006). In such contexts, a strict and cautious application of the criteria may not be the best approach. Instead, weighing the expected benefits against possible anticipated harms may guide physicians and administrators towards screening, rather than not. Here, personal judgments made about individual circumstances are arguably as valid as strict criteria and formulas. This is perhaps highlighted by recent research where 40 years on, individuals diagnosed and treated for PKU in New Zealand still see themselves as part of a ‘living experiment’ with no known ultimate outcomes (Frank et al. 2007)

## The opportunity cost of the proposed screening

The ethical issue behind some criticisms of newborn screening pivots around the ‘Justice Principle’ (Bailey and Murray 2008; Rawls 1971, 2001), which emphasizes the distribution of risks and benefits across populations in an equitable fashion. Here, the argument is that better health gains might be obtained by investing financial resources in other parts of the health system, and is implicated in the ninth criteria outlined by Wilson and Jungner (1968). Namely, the costs of diagnosis and treatment for specific conditions should be financially weighed against potential medical expenditure, as a whole. Whilst the wise use of resources is an important political and ethical consideration, it can be applied in such an overly simplistic way that important medical interventions and programmes are excluded as funding priorities.

The counterbalancing argument within the Justice Principle is that cases with serious impact and severe outcomes also need special consideration. Treating like cases alike can be rephrased as treating unequal cases unequally. That is, different criteria might apply, or different weighting given within criteria, for unusual situations that do not fit typical scenarios. This may lead to prioritization for the most serious and urgent situations, rather than to the widest spread of health gains across a population.

Submissions from the Access to Medicines Coalition (2007) to the Ministry of Health on the development of a medicine strategy for New Zealand provides a valuable discussion on this issue. The submission from the Access to Medicines Coalition to the Ministry of Health on the development of a medicine strategy for New Zealand. The core of the counterargument is that utilitarian analysis needs a certain level of sophistication, and it must incorporate social context and community values to be a useful tool for analysis and decision making.

Without the additional dimension of social and community values, a rather crude utilitarian analysis that takes a whole population approach might favour widely distributed health gains for the maximum number of people. By contrast, a sophisticated utilitarian analysis might tend to favour those most at risk of severe consequences, with urgency of need influencing how priorities are set, thus providing special consideration in special circumstances.

This approach is well established in emergency care. It is also reflected in New Zealand health policy, with priority given to the health needs of Maori and other population groups. It can arguably be an appropriate consideration for rare diseases that have fatal or severely disabling impacts. However, we note that neither the WHO nor the New Zealand screening criteria provide guidance on this point.

## Screening for later onset and untreatable childhood diseases

Late onset and untreatable conditions directly violate the third and fourth criteria outlined by Wilson and Jungner (1968), with neither readily identifiable symptoms nor adequate treatment options. While proposals to screen for such diseases might be readily rejected at first glance, there are valid reasons for giving them serious consideration in the newborn context. The potential negative aspects are the affront to autonomy and apparent lack of benefits for the baby in gaining knowledge that might appear to bring only harm, and the denial of ordinary life experiences unencumbered by the certainty of impending disease impacts.

Many critics of expanded screening argue that neonatal screening can only be justified if the child (the person to be screened) can be expected to benefit directly, and such a view would exclude expansion into conditions that appear later in childhood and which may have limited treatment options. In contrast, within patient/family advocacy groups, there has been widespread discussion about the opportunity to learn about an inherited disease in a child, prior to the birth of a second or third child who might potentially be affected (Wilcken 2012). Another perceived benefit is avoiding the “diagnostic odyssey” associated with complex diseases that present with subtle symptoms in the first months or years. This odyssey can be particularly stressful for families as uncertainty and possibly incorrect diagnosis and inappropriate interventions are experienced.

When advances in screening technologies indicate that particular diseases may be candidates for newborn testing, the associated benefits for affected families provide a significant argument for their consideration. Prime examples are lysosomal storage diseases and Fragile X syndrome. Both of these disease groups frequently present with subtle or minimal symptoms for several years, and when a second or third child is born before the first is diagnosed, families with two or more affected children are certainly not exceptions in our society.

Within advocacy groups, the arguments are well rehearsed, including the principle that ‘benefit to the family is also a benefit to the child’. The policy statement of the Human Genetics Society of Australasia (2011) and the American College of Medical Geneticists (Burchbinder and Timmermans 2011) also makes explicit reference to this principle. However, these societies are in a minority of professional groups that clearly articulate this point. As these examples demonstrate the WHO criteria can be critiqued using a grounded approach, hence providing an argument for newborn screening of particular rare disorders. As we have argued, this has already occurred in practice within the New Zealand context. However, a notable feature of some critics’ arguments is the potential for harm associated with the early identification of a disease, serving as a reason not to add them to a screening programme. This has led to a ‘do nothing’ approach where such potential harms are perceived, without problematizing the consequences of not acting (Pollitt 2006).

As Pollitt (2006) notes, despite the possible harm of expanding too soon without detailed evidence and data, there can also be substantial costs in harms from a ‘do nothing’ approach. A challenge to that approach may come from the ethical framework proposed by Bernheim et al. (2007). This three-part framework proposes an analysis of any ethical issues, followed by an evaluation of the ethical dimensions of alternative actions, and after weighing the two against one another, the provision of justification for any action to be taken. This framework would offer a chance for wider considerations of actions and consequences, leaving aside a strict rule-based approach and allowing a wider range of factors to be considered. Our observations suggest that this is what has happened in practice when some innovations in newborn screening have been decided upon.

## Public policy: ethics, rights and duty

‘Respect for persons’ is more than simply a focus on autonomy, consent and protection of the individual’s interests. In today’s world, it means direct stakeholder involvement in system planning and decision making. As the New Zealand case study has demonstrated, in the context of newborn screening, it should also mean factoring in the family’s interests into the criteria outlined in policy documents. Examples of the application of such criteria to related areas that we are familiar with include: genetic services staff debating the genetic testing of siblings and an HGSA ethics committee considering policies on the genetic testing of minors.

Observation of the processes and reading literature on the topic suggest that for some involved in screening policy and practice, the criteria they work to can sometimes become an end in themselves. In contrast to the criticism often leveled at families, that they are too emotional or subjective in their approach to such issues, some policy makers may be, ironically, too “close” to the administrative and economic issues at hand and the “formula” that often evolves from the criteria to be sufficiently objective. Furthermore, they may also be too far removed from the immediacy of the family and patient experience to be sufficiently subjective, and thus empathetic, in their decision making. With no experience of living on a day-to-day basis with the disorders under consideration, or even unfamiliarity with them, policy officials may lack insight into the implications of their actions for the affected families.

A better blend of decision-making interests that closely involves patient/family interests is required. In New Zealand, such a principle is well supported by provisions in the Public Health and Disability Act 2000, including S3(c) providing for a community voice, and S22 (1), (g), (h) and (i) with their emphasis on social responsibility, community engagement and ethical standards. But the question remains as to how these ethical implications should be factored into decision making.

In response to this question, we propose a pragmatic ethic for consideration, with action in the face of uncertainty or in the face of questionable cost-effectiveness. That is, when knowledge of biological causes and the technical capacity to intervene intersect, professionals and administrators within the health system are faced with an emerging duty to act, and the implicated families/patients have an emerging right to services within the health system. While these duties and rights may not always be absolute, or legally enforceable due to the constraints of competing health demands and limited resources, there is, arguably, still an ethical responsibility for the health system to respond in a timely, considered manner to patient and family needs.

We propose that both the right and the duty are elevated by the seriousness and urgency associated with particular disease groups. Thus, in the context of screening, priority should be given to appropriate assessments of the potential and suitability of a disease, as opposed to the ongoing delays that seem to characterize many potential screening situations. The three-part framework of Bernheim et al. (2007) would seem very apt for this situation.

In the New Zealand context, one such example of an intervention of rights and duty in a policy decision was the Health & Disability Commissioner’s ruling on antenatal HIV screening that occurred in June 2005. The National Health committee considered the case for an antenatal screening programme for HIV and recommended against such a step, but a complaint to the Health and Disability Commissioner resulted in his review of the rights of patients under the Health and Disability Consumers Code of Rights, and concluding:

“Given the state of knowledge about HIV infection and the availability of treatment to prevent perinatal transmission, in my view, women receiving antenatal care in New Zealand in 1999 were entitled to a comprehensive pregnancy risk assessment that included assessment of the risk of HIV infection” (Health and Disability Commissioner 2005).

The comments from the Commissioner relate to a particular set of circumstances, but they may well be as applicable to newborn metabolic screening as they are to antenatal screening. Indeed, they could hold particular significance for many potential screening initiatives around the antenatal and newborn period, as well as those recently implemented, including newborn hearing, antenatal fetal aneuploidy, antenatal HIV and expanded newborn metabolic screening. Most of those were very slow to reach implementation, and it appears that whilst there was a significant level of data and evidence to support their application, in practice, bureaucratic malaise was the major impediment to the start of these programmes.

## Conclusion—a paradigm shift

This article identifies what appears to be a paradigm shift in the implementation of newborn screening. Other authors have noted this, but with varying degrees of acceptance that issues such as the interests of the patient’s family should be part of the decision criteria (Seymour et al. 1997). This participation is supported by the principle of acceptability to those screened, or to those consenting on their behalf, as well as consistency with many other trends in decision making in society.

In the New Zealand context, decisions to implement antenatal HIV screening programmes and cabinet decisions on antenatal Down syndrome screening also demonstrate that formulaic application of screening criteria is not enough (New Zealand Ministry of Health, 2007). Both decisions included other factors and effectively broadened the standard criteria to incorporate wider ethical, legal and social considerations. Such matters are increasingly being acknowledged in the final decision on whether to screen or not. In other jurisdictions, such as some US States’ decisions on a variety of new screening initiatives, wishes of families appear to have significant influence.

While all screening criteria could usefully be reviewed in the light of animated debates about screening practices, newborn metabolic screening criteria in particular need close scrutiny and change in the light of the important social, political and ethical aspects that should be included. In light of our analysis of screening in New Zealand, and from observation of screening literature and practices in other jurisdictions, we propose that for screening in the newborn period, the following additional criteria should apply:

- Screening in the absence of an accepted treatment may be appropriate when it will provide information of benefit to the child or the family.
- Benefit or harm to the family should be considered a benefit or harm to the child.
- Decisions about screening should include community values, rights and duties alongside any cost-effectiveness assessment.
- Action in the face of uncertainty may be justified in exceptional circumstances.

Widening criteria for screening the newborn period, as proposed, will allow a far more accommodating balance of interests, and adapt historic generic screening criteria to reflect contemporary circumstances, knowledge and values, including particularities of the newborn situation.
